# 2-Bromo-4,4-dimethyl-1-(2,4,5-trimethoxy­phen­yl)pentan-3-one

**DOI:** 10.1107/S1600536809018601

**Published:** 2009-05-23

**Authors:** Lin-Lin Tang, Jiao Ye, Qi-Xing Liu, Ai-Xi Hu

**Affiliations:** a College of Chemistry and Chemical Engineering, Hunan University, Changsha 410082, People’s Republic of China

## Abstract

The three meth­oxy groups of the title compound, C_16_H_23_BrO_4_, are almost coplanar with the attached aromatic ring, forming dihedral angles of 7.19 (13), 2.48 (13) and 7.24 (12)°. The crystal structure shows an intra­molecular and an inter­molecular C—H⋯O inter­action.

## Related literature

For background and related structures, see: Xu *et al.* (2007 ▶); Hu *et al.* (2007 ▶).
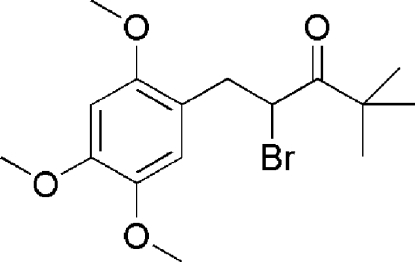


## Experimental

### 

#### Crystal data

C_16_H_23_BrO_4_
*M* *_r_* = 359.25
Triclinic, 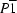

*a* = 9.0173 (5) Å
*b* = 9.2086 (5) Å
*c* = 11.4217 (6) Å
α = 106.752 (1)°
β = 106.196 (1)°
γ = 100.353 (1)°
*V* = 836.51 (8) Å^3^
*Z* = 2
Mo *K*α radiation
μ = 2.47 mm^−1^
*T* = 173 K
0.47 × 0.40 × 0.21 mm

#### Data collection

Bruker SMART 1000 CCD diffractometer
Absorption correction: multi-scan (*SADABS*; Bruker, 2004 ▶) *T*_min_ = 0.370, *T*_max_ = 0.594
6537 measured reflections
3237 independent reflections
2940 reflections with *I* > 2σ(*I*)
*R*_int_ = 0.017

#### Refinement

*R*[*F* ^2^ > 2σ(*F* ^2^)] = 0.025
*wR*(*F* ^2^) = 0.073
*S* = 1.08
3237 reflections
196 parameters
H-atom parameters constrained
Δρ_max_ = 0.65 e Å^−3^
Δρ_min_ = −0.21 e Å^−3^

### 

Data collection: *SMART* (Bruker, 2001 ▶); cell refinement: *SAINT-Plus* (Bruker, 2003 ▶); data reduction: *SAINT-Plus*; program(s) used to solve structure: *SHELXTL* (Sheldrick, 2008 ▶); program(s) used to refine structure: *SHELXTL*; molecular graphics: *SHELXTL*; software used to prepare material for publication: *SHELXTL*.

## Supplementary Material

Crystal structure: contains datablocks I, global. DOI: 10.1107/S1600536809018601/bt2955sup1.cif

Structure factors: contains datablocks I. DOI: 10.1107/S1600536809018601/bt2955Isup2.hkl

Additional supplementary materials:  crystallographic information; 3D view; checkCIF report

## Figures and Tables

**Table 1:** Hydrogen-bond geometry (Å, °)

*D*—H⋯*A*	*D*—H	H⋯*A*	*D*⋯*A*	*D*—H⋯*A*
C2—H2⋯O4	1.00	2.50	3.089 (2)	117
C16—H16*B*⋯O2^i^	0.98	2.57	3.465 (3)	151

Symmetry code: (i) 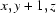.

**Fig. 1.**
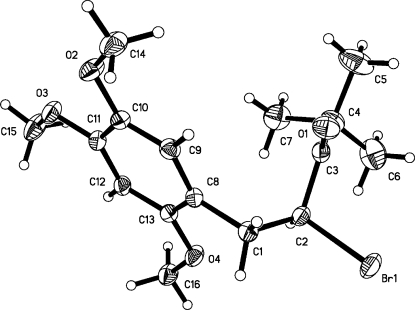
Molecular structure showing 30% probability displacement ellipsoids.

**Fig. 2.**
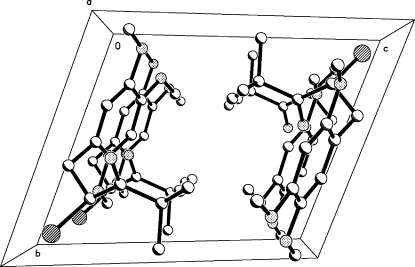
A packing diagram for the title compound. H atoms bonded to C atoms have been omitted for clarity.

## supplementary crystallographic information

### Comment

α-Bromoketone are well known for its universal applications in medical industry and play a key role in the synthesis of thiazole derivatives (Xu *et al., *2007, Hu *et al., *2007). It is found that α-bromoketones work as perfect intermediates and increase the efficiency. Herein we report the synthesis and structure of 2-bromo-4,4-dimethyl-1-(2,4,5-trimethyphenyl)- pentan-3-one.

### Experimental

To the compound of 4,4-dimethyl-1-(2,4,5-trimethyphenyl)pentan-3-one (0.02 mol), 1-butyl-3-methylimidazolidine bromide (0.02 mol) was slowly added. The reaction mixture was stirred for 30 min, then it was extracted with ethyl acetate (30 ml×3). The organic layers were collected, washed with water (20 ml), dried with anhydrous Na_2_SO_4_ and concentrated to give the desired product. Yield: 90.5%. m.p. 366~369 K. ^1^H NMR (CDCl_3_, 600 MHz) δ: 1.01 (s, 9H, 3×CH_3_), 3.20~3.28 (m, 2H, CH_2_), 3.80 (s, 3H, OCH_3_), 3.85 (s, 3H, OCH_3_), 3.87 (s, 3H, OCH_3_), 4.92~4.96 (m, 1H, CHBr), 6.49 (d, J = 4.2 Hz, 1H, C_6_H_2_ 3-H), 6.61 (d, J = 6.0 Hz, 1H, C_6_H_2_ 6-H).

Crystals suitable for X-ray structure determination were obtained by slow evaporation of an ethanol solution at room temperature.

### Refinement

The H-atoms were positioned geometrically, with C—H = 0.95 Å for aromatic, C—H = 0.98 Å for methyl, C—H = 0.99 Å for methylene and were refined as riding with *U*_iso_(H) = 1.2 or 1.5 *U*_eq_(C_methyl_).

### Crystal data

**Table:**

C_16_H_23_BrO_4_	*Z* = 2
*M**_r_* = 359.25	*F*(000) = 372
Triclinic, *P*1	*D*_x_ = 1.426 Mg m^−^^3^
Hall symbol: -P 1	Melting point = 366–369 K
*a* = 9.0173 (5) Å	Mo *K*α radiation, λ = 0.71073 Å
*b* = 9.2086 (5) Å	Cell parameters from 4833 reflections
*c* = 11.4217 (6) Å	θ = 2.4–27.0°
α = 106.752 (1)°	µ = 2.47 mm^−^^1^
β = 106.196 (1)°	*T* = 173 K
γ = 100.353 (1)°	Block, colorless
*V* = 836.51 (8) Å^3^	0.47 × 0.40 × 0.21 mm

### Data collection

**Table:**

Bruker SMART 1000 CCD diffractometer	3237 independent reflections
Radiation source: fine-focus sealed tube	2940 reflections with *I* > 2σ(*I*)
graphite	*R*_int_ = 0.017
ω scans	θ_max_ = 26.0°, θ_min_ = 2.0°
Absorption correction: multi-scan (*SADABS*; Bruker, 2004)	*h* = −11→11
*T*_min_ = 0.370, *T*_max_ = 0.594	*k* = −11→11
6537 measured reflections	*l* = −14→11

### Refinement

**Table:**

Refinement on *F*^2^	Primary atom site location: structure-invariant direct methods
Least-squares matrix: full	Secondary atom site location: difference Fourier map
*R*[*F*^2^ > 2σ(*F*^2^)] = 0.025	Hydrogen site location: inferred from neighbouring sites
*wR*(*F*^2^) = 0.073	H-atom parameters constrained
*S* = 1.08	*w* = 1/[σ^2^(*F*_o_^2^) + (0.0411*P*)^2^ + 0.3147*P*] where *P* = (*F*_o_^2^ + 2*F*_c_^2^)/3
3237 reflections	(Δ/σ)_max_ = 0.002
196 parameters	Δρ_max_ = 0.65 e Å^−^^3^
0 restraints	Δρ_min_ = −0.21 e Å^−^^3^

### Special details

**Table:**

Geometry. All e.s.d.'s (except the e.s.d. in the dihedral angle between two l.s. planes) are estimated using the full covariance matrix. The cell e.s.d.'s are taken into account individually in the estimation of e.s.d.'s in distances, angles and torsion angles; correlations between e.s.d.'s in cell parameters are only used when they are defined by crystal symmetry. An approximate (isotropic) treatment of cell e.s.d.'s is used for estimating e.s.d.'s involving l.s. planes.
Refinement. Refinement of *F*^2^ against ALL reflections. The weighted *R*-factor *wR* and goodness of fit *S* are based on *F*^2^, conventional *R*-factors *R* are based on *F*, with *F* set to zero for negative *F*^2^. The threshold expression of *F*^2^ > σ(*F*^2^) is used only for calculating *R*-factors(gt) *etc*. and is not relevant to the choice of reflections for refinement. *R*-factors based on *F*^2^ are statistically about twice as large as those based on *F*, and *R*- factors based on ALL data will be even larger.

### Fractional atomic coordinates and isotropic or equivalent isotropic displacement parameters (Å^2^)

**Table:**

	*x*	*y*	*z*	*U*_iso_*/*U*_eq_	
Br1	1.06994 (2)	0.86479 (2)	0.12181 (2)	0.03353 (9)	
C1	0.8126 (2)	0.6005 (2)	0.07781 (19)	0.0234 (4)	
H1A	0.7458	0.6459	0.0216	0.028*	
H1B	0.8596	0.5309	0.0243	0.028*	
C2	0.9469 (2)	0.7329 (2)	0.19069 (19)	0.0216 (4)	
H2	0.8996	0.7993	0.2477	0.026*	
C3	1.0691 (2)	0.6722 (2)	0.27311 (19)	0.0224 (4)	
C4	1.1235 (3)	0.7470 (3)	0.4214 (2)	0.0310 (5)	
C5	1.2550 (3)	0.6801 (4)	0.4841 (3)	0.0517 (7)	
H5A	1.2123	0.5653	0.4571	0.078*	
H5B	1.2899	0.7282	0.5794	0.078*	
H5C	1.3468	0.7039	0.4560	0.078*	
C6	1.1885 (3)	0.9271 (3)	0.4661 (3)	0.0466 (6)	
H6A	1.2815	0.9533	0.4399	0.070*	
H6B	1.2213	0.9734	0.5613	0.070*	
H6C	1.1042	0.9696	0.4256	0.070*	
C7	0.9764 (3)	0.7038 (3)	0.4612 (2)	0.0399 (5)	
H7A	0.8945	0.7514	0.4249	0.060*	
H7B	1.0095	0.7439	0.5566	0.060*	
H7C	0.9316	0.5886	0.4274	0.060*	
C8	0.7081 (2)	0.5042 (2)	0.12787 (18)	0.0214 (4)	
C9	0.7162 (2)	0.3520 (2)	0.12133 (19)	0.0230 (4)	
H9	0.7914	0.3107	0.0881	0.028*	
C10	0.6174 (2)	0.2603 (2)	0.16207 (19)	0.0223 (4)	
C11	0.5060 (2)	0.3213 (2)	0.21148 (19)	0.0222 (4)	
C12	0.4989 (2)	0.4740 (2)	0.22142 (19)	0.0222 (4)	
H12	0.4255	0.5165	0.2565	0.027*	
C13	0.5997 (2)	0.5642 (2)	0.17981 (19)	0.0221 (4)	
C14	0.7250 (3)	0.0427 (3)	0.1048 (2)	0.0342 (5)	
H14A	0.6990	0.0390	0.0145	0.051*	
H14B	0.7131	−0.0648	0.1058	0.051*	
H14C	0.8361	0.1074	0.1563	0.051*	
C15	0.3096 (3)	0.2861 (3)	0.3105 (3)	0.0386 (5)	
H15A	0.3760	0.3772	0.3897	0.058*	
H15B	0.2506	0.2054	0.3341	0.058*	
H15C	0.2330	0.3198	0.2520	0.058*	
C16	0.4788 (2)	0.7768 (2)	0.2246 (2)	0.0292 (4)	
H16A	0.3724	0.7047	0.1671	0.044*	
H16B	0.4866	0.8811	0.2172	0.044*	
H16C	0.4939	0.7862	0.3151	0.044*	
O1	1.11572 (18)	0.56644 (17)	0.21908 (15)	0.0306 (3)	
O2	0.61793 (17)	0.11030 (16)	0.15966 (15)	0.0291 (3)	
O3	0.41020 (17)	0.22188 (16)	0.24643 (15)	0.0283 (3)	
O4	0.60042 (16)	0.71626 (16)	0.18717 (15)	0.0281 (3)	

### Atomic displacement parameters (Å^2^)

**Table:**

	*U*^11^	*U*^22^	*U*^33^	*U*^12^	*U*^13^	*U*^23^
Br1	0.03262 (13)	0.02892 (13)	0.05128 (16)	0.00848 (9)	0.02332 (10)	0.02369 (10)
C1	0.0223 (9)	0.0241 (9)	0.0231 (10)	0.0050 (7)	0.0070 (8)	0.0096 (8)
C2	0.0217 (9)	0.0201 (9)	0.0271 (10)	0.0059 (7)	0.0119 (8)	0.0113 (8)
C3	0.0191 (9)	0.0221 (9)	0.0260 (10)	0.0044 (7)	0.0089 (8)	0.0089 (8)
C4	0.0303 (11)	0.0362 (12)	0.0252 (11)	0.0122 (9)	0.0077 (9)	0.0094 (9)
C5	0.0510 (15)	0.081 (2)	0.0325 (13)	0.0360 (15)	0.0117 (12)	0.0258 (13)
C6	0.0446 (14)	0.0386 (13)	0.0362 (13)	0.0023 (11)	0.0065 (11)	−0.0025 (10)
C7	0.0466 (14)	0.0497 (14)	0.0325 (12)	0.0184 (11)	0.0215 (11)	0.0176 (11)
C8	0.0175 (8)	0.0228 (9)	0.0216 (9)	0.0027 (7)	0.0045 (7)	0.0088 (7)
C9	0.0213 (9)	0.0241 (9)	0.0237 (10)	0.0076 (7)	0.0076 (8)	0.0085 (8)
C10	0.0228 (9)	0.0183 (9)	0.0248 (10)	0.0059 (7)	0.0053 (8)	0.0091 (7)
C11	0.0197 (9)	0.0211 (9)	0.0231 (9)	0.0021 (7)	0.0056 (8)	0.0083 (7)
C12	0.0170 (9)	0.0219 (9)	0.0272 (10)	0.0045 (7)	0.0076 (8)	0.0088 (8)
C13	0.0194 (9)	0.0190 (9)	0.0261 (10)	0.0044 (7)	0.0043 (8)	0.0093 (7)
C14	0.0376 (12)	0.0253 (10)	0.0515 (14)	0.0165 (9)	0.0242 (11)	0.0175 (10)
C15	0.0423 (13)	0.0280 (11)	0.0578 (15)	0.0097 (9)	0.0332 (12)	0.0182 (11)
C16	0.0277 (10)	0.0230 (10)	0.0418 (12)	0.0104 (8)	0.0159 (9)	0.0132 (9)
O1	0.0327 (8)	0.0306 (8)	0.0337 (8)	0.0175 (6)	0.0134 (6)	0.0121 (6)
O2	0.0356 (8)	0.0218 (7)	0.0403 (8)	0.0128 (6)	0.0209 (7)	0.0159 (6)
O3	0.0285 (7)	0.0222 (7)	0.0403 (8)	0.0058 (6)	0.0184 (7)	0.0145 (6)
O4	0.0236 (7)	0.0214 (7)	0.0473 (9)	0.0094 (5)	0.0169 (7)	0.0177 (6)

### Geometric parameters (Å, °)

**Table:**

Br1—C2	1.9693 (18)	C8—C9	1.398 (3)
C1—C8	1.514 (3)	C9—C10	1.382 (3)
C1—C2	1.519 (3)	C9—H9	0.9500
C1—H1A	0.9900	C10—O2	1.374 (2)
C1—H1B	0.9900	C10—C11	1.407 (3)
C2—C3	1.536 (3)	C11—O3	1.365 (2)
C2—H2	1.0000	C11—C12	1.392 (3)
C3—O1	1.205 (2)	C12—C13	1.393 (3)
C3—C4	1.525 (3)	C12—H12	0.9500
C4—C5	1.530 (3)	C13—O4	1.377 (2)
C4—C6	1.533 (3)	C14—O2	1.429 (2)
C4—C7	1.542 (3)	C14—H14A	0.9800
C5—H5A	0.9800	C14—H14B	0.9800
C5—H5B	0.9800	C14—H14C	0.9800
C5—H5C	0.9800	C15—O3	1.422 (3)
C6—H6A	0.9800	C15—H15A	0.9800
C6—H6B	0.9800	C15—H15B	0.9800
C6—H6C	0.9800	C15—H15C	0.9800
C7—H7A	0.9800	C16—O4	1.428 (2)
C7—H7B	0.9800	C16—H16A	0.9800
C7—H7C	0.9800	C16—H16B	0.9800
C8—C13	1.393 (3)	C16—H16C	0.9800
			
C8—C1—C2	110.66 (16)	C13—C8—C9	118.31 (17)
C8—C1—H1A	109.5	C13—C8—C1	120.84 (17)
C2—C1—H1A	109.5	C9—C8—C1	120.84 (17)
C8—C1—H1B	109.5	C10—C9—C8	121.62 (18)
C2—C1—H1B	109.5	C10—C9—H9	119.2
H1A—C1—H1B	108.1	C8—C9—H9	119.2
C1—C2—C3	112.96 (15)	O2—C10—C9	125.42 (17)
C1—C2—Br1	109.43 (13)	O2—C10—C11	115.27 (17)
C3—C2—Br1	105.97 (12)	C9—C10—C11	119.30 (17)
C1—C2—H2	109.5	O3—C11—C12	124.48 (17)
C3—C2—H2	109.5	O3—C11—C10	115.70 (16)
Br1—C2—H2	109.5	C12—C11—C10	119.82 (17)
O1—C3—C4	122.68 (18)	C11—C12—C13	119.80 (17)
O1—C3—C2	119.30 (17)	C11—C12—H12	120.1
C4—C3—C2	118.00 (16)	C13—C12—H12	120.1
C3—C4—C5	109.50 (18)	O4—C13—C12	123.22 (17)
C3—C4—C6	110.49 (19)	O4—C13—C8	115.67 (17)
C5—C4—C6	109.6 (2)	C12—C13—C8	121.11 (17)
C3—C4—C7	107.65 (17)	O2—C14—H14A	109.5
C5—C4—C7	109.5 (2)	O2—C14—H14B	109.5
C6—C4—C7	110.09 (19)	H14A—C14—H14B	109.5
C4—C5—H5A	109.5	O2—C14—H14C	109.5
C4—C5—H5B	109.5	H14A—C14—H14C	109.5
H5A—C5—H5B	109.5	H14B—C14—H14C	109.5
C4—C5—H5C	109.5	O3—C15—H15A	109.5
H5A—C5—H5C	109.5	O3—C15—H15B	109.5
H5B—C5—H5C	109.5	H15A—C15—H15B	109.5
C4—C6—H6A	109.5	O3—C15—H15C	109.5
C4—C6—H6B	109.5	H15A—C15—H15C	109.5
H6A—C6—H6B	109.5	H15B—C15—H15C	109.5
C4—C6—H6C	109.5	O4—C16—H16A	109.5
H6A—C6—H6C	109.5	O4—C16—H16B	109.5
H6B—C6—H6C	109.5	H16A—C16—H16B	109.5
C4—C7—H7A	109.5	O4—C16—H16C	109.5
C4—C7—H7B	109.5	H16A—C16—H16C	109.5
H7A—C7—H7B	109.5	H16B—C16—H16C	109.5
C4—C7—H7C	109.5	C10—O2—C14	116.49 (15)
H7A—C7—H7C	109.5	C11—O3—C15	116.98 (15)
H7B—C7—H7C	109.5	C13—O4—C16	118.02 (15)
			
C8—C1—C2—C3	66.5 (2)	O2—C10—C11—O3	−2.0 (2)
C8—C1—C2—Br1	−175.74 (12)	C9—C10—C11—O3	178.37 (17)
C1—C2—C3—O1	43.4 (2)	O2—C10—C11—C12	178.26 (17)
Br1—C2—C3—O1	−76.38 (19)	C9—C10—C11—C12	−1.4 (3)
C1—C2—C3—C4	−135.17 (18)	O3—C11—C12—C13	−178.37 (18)
Br1—C2—C3—C4	105.03 (17)	C10—C11—C12—C13	1.4 (3)
O1—C3—C4—C5	6.3 (3)	C11—C12—C13—O4	−179.61 (17)
C2—C3—C4—C5	−175.18 (19)	C11—C12—C13—C8	0.1 (3)
O1—C3—C4—C6	127.1 (2)	C9—C8—C13—O4	178.22 (17)
C2—C3—C4—C6	−54.3 (2)	C1—C8—C13—O4	−2.7 (3)
O1—C3—C4—C7	−112.6 (2)	C9—C8—C13—C12	−1.5 (3)
C2—C3—C4—C7	65.9 (2)	C1—C8—C13—C12	177.52 (18)
C2—C1—C8—C13	74.0 (2)	C9—C10—O2—C14	−3.0 (3)
C2—C1—C8—C9	−107.0 (2)	C11—C10—O2—C14	177.39 (18)
C13—C8—C9—C10	1.5 (3)	C12—C11—O3—C15	−7.3 (3)
C1—C8—C9—C10	−177.55 (18)	C10—C11—O3—C15	172.93 (18)
C8—C9—C10—O2	−179.66 (18)	C12—C13—O4—C16	−7.7 (3)
C8—C9—C10—C11	−0.1 (3)	C8—C13—O4—C16	172.52 (17)

### Hydrogen-bond geometry (Å, °)

**Table:**

*D*—H···*A*	*D*—H	H···*A*	*D*···*A*	*D*—H···*A*
C2—H2···O4	1.00	2.50	3.089 (2)	117
C16—H16B···O2^i^	0.98	2.57	3.465 (3)	151

Symmetry codes: (i) *x*, *y*+1, *z*.
